# Manual Cultivation Operations in Poplar Stands: A Characterization of Job Difficulty and Risks of Health Impairment

**DOI:** 10.3390/ijerph16111911

**Published:** 2019-05-30

**Authors:** Tiberiu Marogel-Popa, Marius Cheţa, Marina Viorela Marcu, Cristian Ionuţ Duţă, Florin Ioraş, Stelian Alexandru Borz

**Affiliations:** 1 Department of Forest Engineering, Forest Management Planning and Terrestrial Measurements, Faculty of Silviculture and Forest Engineering, Transilvania University of Braşov, Şirul Beethoven No. 1, 500123 Braşov, Romania; marogel_tibi@yahoo.com (T.M.-P.); marius.cheta@unitbv.ro (M.C.); viorela.marcu@unitbv.ro (M.V.M.); duta_cristianionut@yahoo.com (C.I.D.); 2 Buckinghamshire New University, Queen Alexandra Road, High Wycombe, Buckinghamshire HP11 2JZ, UK; florin.ioras@bucks.ac.uk

**Keywords:** manual cultivation, job characterization, ergonomics, efficiency, cardiovascular workload, work intensity, risk of musculoskeletal disorders

## Abstract

Short rotation poplar forests are a viable alternative in producing high quality wood for industrial applications. Their success depends on timely and high-quality implementation of a series of operations. Weed control operations are implemented to favor the trees in their competition for soil resources, and cultivation is an option typically used in many European countries. For the moment, a complete mechanization of such operations is virtually impossible, and they still require an intensive use of manual labor. Since information on work difficulty and risks in manual cultivation operations is limited, this study aimed to characterize this job. Evaluation was made in terms of work efficiency, cardiovascular workload, work intensity and postural risks by implementing a time and motion study combined with heart rate measurements, accelerometry and whole-body postural analysis. Work efficiency was particularly low even if the share of effective work time was high (70% of the observation time). Job was characterized as moderate to high intensity, which resulted into a moderate to high cardiovascular strain. While the postural analysis indicated rather small risks, the main problem was found for the back postures assumed during the work. Improvements should aim to extend mechanization, train the workers and appropriately design rest breaks.

## 1. Introduction

Short rotation cultivated forests are considered to be a valuable alternative for wood provisioning, enabling the possibility to reduce the anthropogenic disturbance on natural forests and to conserve them. To enable a timely provisioning of wood to industry, such forests need to be cultivated using fast-growing trees able to provide high-quality wood. Among the existing fast-growing species, poplars are intensively used in many countries for such outcomes [1]. Their cultivation supposes a sequence of operations including fertilization, irrigation and weed control [2], with the last one aiming to balance the competition for soil resources and being carried out, in many regions, by herbicide application, cultivation or a combination of the two [1]. Some of these practices (i.e., in Romania) are used even in regular poplar forests that could be easily assimilated to short rotation cultures due to the propagation techniques and geometrical plantation schemes that are used, types of implemented operations and rotation length. In such conditions, the typical way of carrying on the weed control is by cultivation.

The level of mechanization in forest operations depends on many factors such as the forest type, wood species, management methods, terrain and climatic conditions [3], with many of the Eastern European countries using operational systems that are partly mechanized [4]. This is particularly the case of forest establishment [5] and cultivation operations [6] that are still requiring manual labor to a great extent. In addition, many of the forest work places are characterized by difficult operational conditions and the work in forest itself is seen to be amongst the most difficult and hazardous jobs [7]. Since many of forest operations still require manual work, their engineering and management requires, at least in a first stage, their understanding in terms of difficulty and hazards. Based on such knowledge, work (re)design may be employed to ensure that work tasks are aligned to human capability, by measures designed to prevent adverse health effects [8] that should be further related to several key areas of the general work system such as the risk assessment, accountability awareness, physical and mental workload, quality of work environment and work technology [9].

Manual cultivation operations have received less attention in ergonomic assessments of forest operations which are dominated by research on harvesting operations [10]. As a fact, only one study [11] was found in the available literature dealing with similar problems; it concluded that manual weed control is a highly demanding job from a physiological point of view, exposing the workers to increased cardiovascular workloads [11]. In the Romanian practice, manual cultivation operations of poplar forests are typically coupled with mechanized ones, in a double-pass system in which the mechanized equipment is operating on the space available between the rows of trees in such a manner that enables the protection of aerial tree parts; the rest of area is approached by workers equipped with hoes [6].

Given the limited information availability on the difficulty and risks of such jobs, the main aim of this study was to describe, document and characterize manual cultivation operations from an ergonomic point of view, to be able to draw conclusions and implications for the public health associated with this occupation. Since the ergonomics and public health cover many key sub-disciplines, it was virtually impossible to approach all the inter-relations between the workers, their job tasks and the operational environment.

To this end, musculoskeletal disorders (MSDs) are the most common cause of severe long-term pain and physical disability, and they affect hundreds of millions of people around the world. The role of psychosocial factors and work-related stress in the development of MSDs has received increased attention. Indeed, a number of epidemiological studies have been conducted in different sectors (from office work to manual work), repeatedly showing linkages between work-related psychosocial factors and MSDs. Overall, it is evident that the incidence of MSDs is associated with high perceived work-related stress levels, high workload and demands, and monotonous work [12], that may lead to public health problems related to different kinds of occupations, highlighting serious problems associated with the forest operations industry [13] which, in turn, may lead to significant temporary or permanent disability of workers [14]. 

In regard to manual cultivation operations, the initial assumptions of this study were that the work productivity would be particularly low and the workplace time would be characterized by an increased proportion of the time spent in rest pauses and delays, the experienced work difficulty would be particularly high given the characteristics of the tasks, the job itself would be characterized by a high intensity of dynamic work, as well as the assumed postures would generate risks for the upper limbs of the body, in particular for the back. For this reason, the study focused on: (i) characterizing the work performance by a typical time and motion study, (ii) describing the physiological workload in terms of cardiovascular activity, (iii) evaluating the intensity of work by body movement benchmarking techniques and (iv) assessing the risks of musculoskeletal disorders by a postural assessment method.

## 2. Materials and Methods

### 2.1. Study Locations, Forest Condition and Study Subjects

Three study locations (Table 1) were chosen in the southeastern part of Romania, close to the Danube river, in the forests managed by three forest districts. The first study location (L1) was selected in the Management Unit II Ciuperceni, compartment no. 88D managed by Forest District of Calafat where the observations were carried out in two days: 13rd and 22nd of June 2018. The second location (L2) was selected from the forests managed by Forest District of Segarcea (Management Unit I, compartment no. 6C) and the third location (L3) was selected from the forests managed by Forest District of Poiana Mare (Management Unit IV Rast, compartment no. 70A). In L2, field observations were carried out in 18th of June 2018, while in L3 they were extended on 3 days (19th to 21st of June 2018). Location selection in the field was based on criteria such as the current practices used to establish the forests, job availability in given areas and the dimensional variability of weed to be removed by manual cultivation.

In all of the selected locations, hybrid poplar (*Populus × euroamericana* (Dode) Guinier) [15] forests were established by artificial regeneration (plantation). The forest in L1 was established in 2013 by a 4 × 4 m plantation scheme, while the forests from L2 and L3 were established in 2015 and 2017 respectively, using a 5 × 4 m plantation scheme. Both, plantation and cultivation operations of poplar forests in the area are carried out using locally available workers who are quite experienced in such operations given their background in similar agricultural practices. A number of 14 male workers (hereafter subjects) having an extended experience in regular farming, including cultivation operations, were selected from the local population based on their verbal and informed consent to participate as anonymous subjects in the study. The goal of the study, the intended use of data as well as the procedures required by the study were detailly explained to each of them in advance and they were instructed to carry on their jobs as they are used to. Given the limited availability of monitoring devices (3 sets of devices), form these workers, three subjects were randomly sampled each day and for each location for a detailed monitoring of work.

The sample of workers taken into study was characterized by an age of 46.4 ± 14.0 years, a body weight of 82.94 ± 15.43 kg and a height of 174.5 ± 5.9 cm (Table 2), being representative for the population of workers from the study area which, in many cases is quite aged.

### 2.2. Work Layout

**Figure 1:**
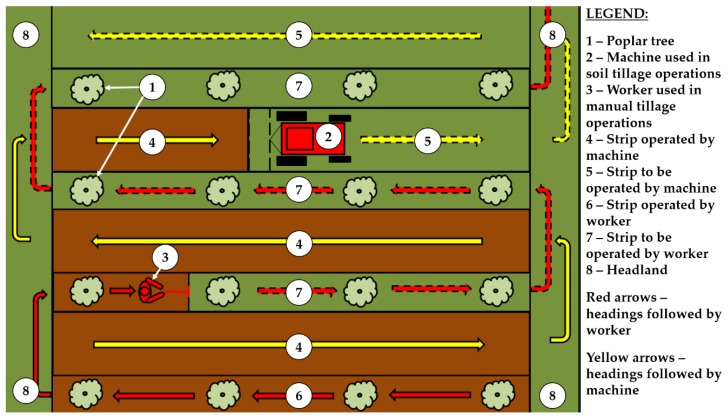
Operational layout (concept) used for cultivation operations in the area taken into study.

In the Romanian practice related to hybrid poplar forests, cultivation operations are typically implemented using a two-pass operational system. In a first step, machines such as tractors equipped with mowers, ploughs or harrows are used for cultivation operations on a single direction of the operated plots to mobilize the soil and to remove the weed between rows (Figure 1). The remaining strips which contain the trees are manually operated in a second pass, by teams of manual workers using hoes. In this operational configuration, the local practice makes use of mechanization for approximately 80% of the area while the rest is operated by manual means.

Nevertheless, depending on the plantation scheme and spaces existing between the tree rows, on one hand, and on the width of equipment attached to tractors, on the other hand, some cases require more than one inter-row tractor pass. It was the case of this study, where the inter-row area was covered by more than one mechanized pass, following that, on each tree row, the area to be operated by manual means to account for approximately one meter in width. For the manual operations, which made the scope of this study, the work organization was rather simple: each worker entered one row at the headland, operated the row and reentered a new row at the opposite headland. Therefore, the work was divided for further analysis based on the tasks observed in the field such as the effective work (EW) consisting of manual cultivation, rest pauses (RP) consisting of all the breaks taken by subjects in the field to rest, meal pauses (MP) and delays (D) which included the delays caused by the study itself and some minor technical delays. During the study, the sky was partly clouded and the air temperature (Table 1) was considered to be low enough to exclude the thermal stress effects on the subjects (e.g., [16]), given the fact that the locations were partly shaded by the surrounding mature forests.

### 2.3. Data Collection Procedures

In each study location and for each day, the operations were monitored by video recording using a digital camera placed on a tripod at the closest headland and set to continuously record video files of 20 min in length each. The camera was placed is such a manner that enabled the best field of view on the collected files and covered all the three workers monitored in a given day. As the work progressed on the rows, the location of the camera was changed accordingly to be able to keep the needed details visible in its field of view. Data collected this way was used to document the observed work tasks, to extract the time consumption on tasks and to evaluate the cardiovascular workload and the risks of musculoskeletal disorders by a postural analysis implemented in the office phase of the study. The height and the width of the weed stratum was visually evaluated and noted into a field book along with the main anthropometric characteristics of the observed subjects such as the age (A, years), body weight (W, kilograms) and height (H, centimeters), with the last two being used to compute the body mass index (BMI, Table 2) using its specific formula.

Polar ^®^ V800 dataloggers (Polar, Kempele, Finland) including their H7 heart monitoring sensors mounted on straps were used to monitor the subjects’ cardiovascular activity during the undertaken tasks in terms of heart rate (HR, beats per minute). Procedures used to estimate the heart rate at rest (HRr, beats per minute), setup, collect, download and pre-process the data including that referring to location, were similar to those described in [17]. Data needed to evaluate the intensity of work (WI) was collected by the means of new, factory-calibrated, tri-axial accelerometers—Extech ^®^ VB300 (Extech Instruments, FLIR Commercial Systems Inc., Nashua, NH, USA) attached to the pericardial strap of the heart rate datalogger. The devices were placed on the back of each subject in between scapulae, as close as possible to the middle of spine’s thoracic vertebrae section. The choice of this body part was based on the assumption that most of the changes in the acceleration signal, therefore changes in work intensity, will be caused by movements of the subjects’ back, given the characteristics of monitored operations. Procedures used to setup, collect, download and pre-process the raw acceleration data were similar to those described in [18]. Both dataloggers were setup to collect data at one second rate.

The main weather characteristics during the study (air temperature—T, °C and relative humidity—RH, %) were documented as average values for the study periods specific to each observation day. This data was procured from the closest weather station (Calafat, 62 m a.s.l., 43°59′06′′ N–22°56′46′′ E, distance range from study locations of 4 to 56 km).

### 2.4. Data Processing Procedures

Data processing procedures consisted of several steps that were required to obtain the initial databases needed for statistical analysis. Video data was downloaded from digital cameras at the end of each observation day. An initial processing task consisted of a detailed time and motion study that was framed around the concepts used in forest operations [19,20] and which supposed the analysis of video files in their real sequence of observation, followed by data extraction into a Microsoft Excel (Microsoft Excel 2013, Microsoft, Redmond, WA, USA) sheet per time consumption categories, subjects and tasks. To this end, the unit of production (P) in this study was considered to be the manually operated area of one hectare, while the time consumption (t_EW_, t_RP_, t_MP_ and t_D_, seconds) was assumed to belong to the previously identified tasks (EW, RP, MP, D). Given the specificity of this study, only the efficiency metrics were computed (GWER - gross work efficiency rate and NWER - net work efficiency rate, hours/hectare) after time conversion from seconds to hours. The supporting calculation relations are given in Equations (1)–(5):

GWT_i_ (hours) = t_EWi_ (hours) + t_RPi_ (hours) + t_MPi_ (hours) + t_Di_ (hours), (1)

NWT_i_ (hours) = t_EWi_ (hours) + t_RPi_ (hours), (2)

GWER_i_ (hours/ha) = GWT_i_ (hours)/P_i_ (ha), (3)

NWER_i_ (hours/ha) = NWT_i_ (hours)/P_i_ (ha), (4)

P_i_ (ha) = ARW (m) × TRL_i_ (m)/10,000, (5)

where: i stands for a given monitored subject, GWT_i_—gross time of subject i, t_EWi_—effective work time of subject i, t_RPi_—rest pauses time of subject i, t_MPi_—meal pause time of subject i, t_Di_—delay time of subject i, NWT_i_—net time of subject i, GWER_i_—gross work efficiency rate of subject i, P_i_—production of subject i, NWER_i_—net work efficiency rate to subject i, ARW—average row width based on field observation (1 m), TRL_i_—total row length operated by subject i.

The cardiovascular workload of each subject was evaluated at the task, day and location level using the heart rate reserve (%HRR) metric as defined, for instance, in [21]. Acknowledging the usefulness of several other metrics in evaluating the physiological workload in terms of heart activity, the choice of %HRR was based on the limited applicability of average heart rate per tasks to different age groups [22], as well as on the fact that it is expected to be a good predictor of workload only in the range of 100 to 140 beats per minute [16]. Since it was virtually impossible to test the subjects by a preestablished protocol aiming to determine their maximum heart rate (HRmax), the formula HRmax = 220 – age (years) was used to estimate this metric [21]. Procedurally, for each heart rate sample collected in the field, codes were used to document the belonging of each 1-s observation to a given task using as a reference the time labels from heart rate samples and video files respectively.

Tri-axial raw acceleration data was processed in a different way. Assuming that for the same task the intensity of work could vary in a given range, this data was not further documented by codes. Instead, the vector magnitudes for each 1-s observation (Equation (6)) were further processed to exclude the gravity component from the signal using the Euclidian Norm Minus One (ENMO, g) metric (Equation (7)) [23]; then, the resulting, otherwise few and small negative values were converted to zero by a logical function of Microsoft Excel:

(6) $${\mathrm{vm}}_{j}\;(g)=\sqrt{x_{j}^{2}+y_{j}^{2}+z_{j}^{2}},$$

ENMO_j_ (g) = vm_j_ (g) – 1, (7)

where j stands for a given observation, vm_j_ - vector magnitude for observation j, x_j_ - raw response on axis x for observation j, y_j_ - raw response on axis y for observation j, z_j_ - raw response on axis z for observation j, ENMO_j_ - Euclidian Norm Minus One of observation j.

**Figure 2:**
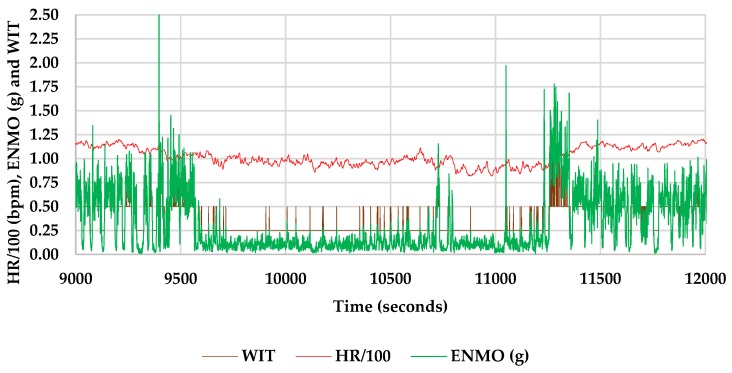
Concept used to separate time epochs for light intensity work (LIW), moderate intensity work (MIW) and high intensity work (HIW). Legend: WIT - work intensity threshold (0.00 to 0.25 for LIW, 0.25 to 1.00 for MIW and more than 1.00 for HIW), HR/100 - heart rate divided by 100 (only for concept demonstration), ENMO—Euclidian Norm Minus One corrected for negative values.

Two work intensity thresholds (WIT) were designed based on the literature documentation to separate the time spent in different work intensities. An ENMO value of less than 0.25 g was used to separate the light intensity work (LIW) and a value of more than 1.00 g was used to separate the high intensity work (HIW) from the datasets collected for each subject. These assumptions were based on the work of [24,25]. Observations falling in the range of 0.25–1.00 g were categorized as moderate intensity work (MIW). Separation and categorization were implemented by simple logical functions applied to the corrected ENMO data in Microsoft Excel (Figure 2).

Risks of musculoskeletal disorders (MSD) were evaluated for each subject, work day and location by the means of Ovako Working posture Analysis System (OWAS) as introduced by Karhu et al. [26], then detailed e.g., [16] and discussed for its applicability in forest operations [7]. The choice of this postural analysis method was based on its history in use in forest operations [17,27,28] capability to analyze the whole body [26,29], simplicity in use [7,26], and possibility to compare the results e.g., [30] including comparisons to those coming from other industries. To this end, each video file collected in the field was broken in frames extracted at 1-s rate. Then, random numbers produced by simple functions in Microsoft Excel were used to extract exactly 100 frames from each video file and for each worker and location of study (Table 3).

This approach resulted in the analysis of 23,700 still images. Those images failing to give in their field of view all the information needed to analyze the whole-body posture of a given subject were considered to be non-valid. Approximately 56% (13,123) of the initial frames were retained as valid and used in statistical analysis (Table 3). Postural analysis was implemented as a detailed analysis of back, arms and legs according to the OWAS method, followed by data coding into Microsoft Excel sheets. Since the force exertion was difficult to evaluate, this component was assumed to be less than 10 kg for each frame, based on the subjective evaluation of researchers that carried out this data processing task. Nevertheless, this approach was consistent with the type and weight of the tools used during the work. Each frame was documented by coding the task to which it belonged, a fact that supposed in some cases some revisions of video files. A Visual Basic for Applications (VBA) logical code was designed to attribute action category (AC) codes for each valid frame. Then, for each worker, day and location, a postural risk index (PRI) was calculated based on the approach described in [28,30]. As an aggregated metric, PRI was used to judge the exposure to risks of developing MSDs. It can take values in between 100 and 400%, where 100% corresponds to AC1, 200% to AC2, 300% to AC3 and 400% to AC4 while the intermediate values need to be judged to choose the appropriate action category.

To enable the characterization of work, data on time consumption, work efficiency, cardiovascular workload, work intensity and postural analysis was aggregated at study level following the statistical analysis.

### 2.5. Statistical Analysis

Right at the beginning of statistical analysis it was evident that the aggregated data coming from each subject working in a given day and location was quite heterogeneous. For this reason, no comparison tests were undertaken to check if there are any differences in terms of work performance outcomes and input resources between subjects, work days and locations. Instead, the statistical techniques used were aligned to the goal of this study that was to characterize the manual cultivation operations as a job. Obviously, this approach needs to include the variability produced by different types of factors [20] such as that given by changes in anthropometric features and human capability, tools used and operational environment conditions. For that, descriptive statistics specific to central tendency were computed and reported. Operational performance in terms of time consumption and efficiency was reported as total time, time shares per work tasks and efficiency rates. Mean values of heart rate reserve were used to characterize the cardiovascular workload per tasks and at the study level while the share of time spent per categories of work intensity was used for similar characterizations. Postural data was computed as shares per action categories at subject and study level, then this data was used to compute the postural risk indexes at subject and study level. Then, a more detailed analysis of back, arms and legs postures was implemented to see what approaches should be taken for work redesign and improvement. To this end, shares of back, arms and legs postures per specific codes were analyzed for all the data taken into study. All of the statistical analyses were carried out in Microsoft Excel.

## 3. Results

### 3.1. Estimates on Time Consumption per Tasks and Operational Performance Metrics

Table 4 shows a breakdown of time consumption and efficiency rates per subjects, days of observations and locations. At study level, field observations were carried out for roughly 85 h. In average, almost 70% of that time was spent as effective work time and approximately 22% was used as rest time. Having meals accounted for approximately 9% of the study time but it was not specific to all the subjects and all the study locations. Other delays, including those caused by the study itself were only minor in the time consumption structure, accounting for less than 1%.

Given the overall distribution of time consumption, net work efficiency rate was estimated at 34.31 h per hectare which was close to gross work efficiency rate (36.35 h per hectare). Since these figures apply to the effective operated area, under real circumstances in which approximately 75–80% of the area is mechanically operated, they will translate into average gross and net efficiency rates in the range of 9.09 to 8.58 h per hectare respectively. 

At subject, observation day and location level, on the other hand, time consumption and efficiency rates figures were rather heterogeneous. The effective work time, for instance, accounted for 45.20 to 83.89% of the observed time, while the rest time varied widely between 13.04 and 54.80%. In general, meal pauses were taken only in those situations in which the total observation time exceeded four hours. Accordingly, the net efficiency rates varied between 14.98 and 69.15 h per hectare while the gross work efficiency rates varied between 16.92 and 62.29 h per hectare.

Given the fact that operational conditions in the three locations were quite different, one could have been expected to find some differences related to that. However, expectations were not entirely met as, for instance, the work performance in L2 was, in average, higher compared to L1, while the height of the weed to be removed was lower in the latter. In this last case, however, the subjects taken into study were characterized by the greatest ages of the sample taken into study (over 45-year-old, most of them over 50).

When comparing the work performance between L2 and L3, one could find that, in average, it was higher in L3, probably due to the better operational conditions but, in general, the work performance was correlated and related to the subject’s age (R = 0.5, R^2^ = 0.26, α = 0.05, *p* < 0.05).

### 3.2. Cardiovascular Workload

In average, the heart rate of the observed subjects varied between 95 (S6) and 126 (S14) beats per minute (Table 5). From this point of view, it seems that S14, in particular, experienced a very heavy work. This may be supported by the greatest share of time spent in rest pauses (Table 4) and by the increased overall heart rate (Table 5).

At the observed sample level, manual cultivation operation seems to be rather a heavy job, taking almost 37% of the heart rate reserve. Rest pauses have not led to a full recovery and to a normal cardiovascular activity (%HRR = 33.6) which is likely not to be reached also during the meal pauses (%HRR = 21.42). Overall, the heart rate reserve was particularly high (%HRR = 35.2) at the observed sample level.

At subject, work day and location level, there was a certain variability in terms of average heart rate, heart rate at rest and heart rate reserve per tasks and per days of observation. Even for the same subject, the average heart rate varied from day to day and from one location to other. Heart rate reserve during the effective work varied between 21.98 and 52.68%, and it was clearly correlated and related to the age of the subjects (R = 0.63, R^2^ = 0.40, α = 0.05, *p* < 0.05). This was true also in the case of the overall heart rate reserve (R = 0.64, R^2^ = 0.40, α = 0.05, *p* < 0.05) which was calculated by taking into account all of the observation time.

In particular, subjects S1, S5, S8, and S10 to S14, accounting for almost 60% of the sample, were those that spent the greatest effort in the observed operations during the effective work. For most of the subjects the effort spent was probably related to their age and less related to the local operational conditions. This was even more so evident as the air temperature of the last observational day was the closest to the thermal comfort (Table 1), the operational conditions were averaged compared to the other two locations (Table 1), while the subjects working there were amongst the oldest in the studied sample (Table 2).

### 3.3. Work Intensity

Tri-axial acceleration dataloggers performed well during the field observation excepting two cases—S10 and S11 working in L1 (Table 6)—where they failed to collect data covering all the observed time. For that reason, data coming from these dataloggers in case of L1 was excluded when characterizing the work intensity at the sample level. Also, some minor differences between the total observed time and the work intensity related survey time were unavoidable since the dataloggers were placed on the workers after starting the camera for video recording. Nevertheless, these differences were only minor.

Shares of time spent in the three work intensity categories is shown in Table 6. At the sample level, roughly 61% of the time was categorized as moderate intensity work and almost 35% were categorized as light intensity work. The share of light intensity work varied between 18.61 (S12 × L1 × 13) and 54.01% (S5 × L3 × 19) while the share of moderate intensity work varied between 43.76 (S5 × L3 × 19) and 75.06% (S12 × L1 × 22). Nearly 5% of the observed data stood for high intensity work. In this last category, the data was quite heterogeneous, with shares between 0.75 (S14) and 9.72 (S13 working in L1).

### 3.4. Postural Risk

**Figure 3:**
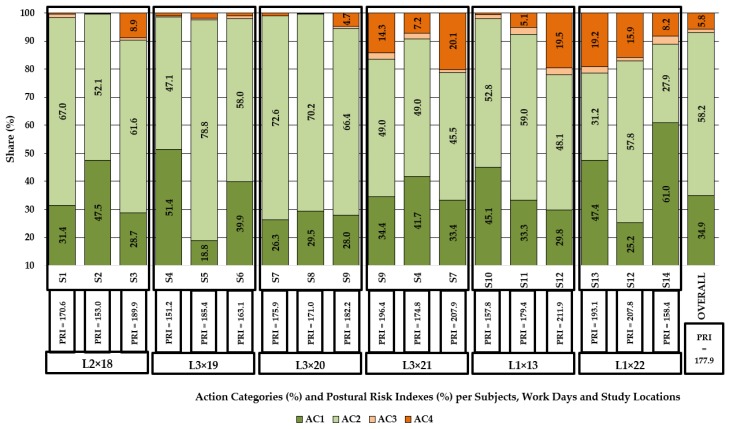
Share of the analyzed frames per action categories and postural risk indexes estimated at subject, location, observation day and sample level.

Figure 3 shows a breakdown per action categories and postural risk indexes estimated at subject, observation day, location and sample level. At sample level, approximately 35% of the analyzed frames were included in the 1st action category, more than half of them were categorized in the 2nd action category and roughly 6% were interpreted as belonging to the 4th action category.

The postural risk index characterizing the job was found to be of almost 178, indicating rather the categorization of this job in the second action category which requires corrective actions to be taken in the near future. At subject level, on the other hand, distribution on action categories and the computed postural risk indexes were quite variable. Frames attributed to the 1st action category accounted for shares of 18.8 to 61%, with the latter one characterizing the postural behavior of S14; frames attributed to the 2nd action category accounted for shares in the range of 27.9–78.8%, those specific to the 3rd action category accounted for minor shares and those belonging to the 4th category accounted for shares of up to 20.1%. The postural risk indexes varied in between 151.2 (S2 × L2 × 18) and 211.9 (S12 × L1 × 13).

At the sample level (Table 7), back postures were found to be particularly uncomfortable, as in more than 55% of the cases, the subjects were found to have the back bent and twisted or bent forward and sideways. Straight postures of the back were found only in 26% of the cases. In general, the arm postures were found to be comfortable and this situation is related to the characteristics and tool use in this kind of job. Combined with poor postures of the back, legs postures codded by 4, 5 and 6 lead always to the worst postural situation which is characteristic to the fourth action category. It was not the case of the analyzed sample since these legs’ postures accounted for only 7%. Therefore, from the postural analysis point of view, the main problems related to potential risks of health impairment were those specific to the back.

## 4. Discussion

The main aim of this work was to characterize the difficulty and risks associated to manual cultivation operations in hybrid poplar forests. Acknowledging the limitation of the results to male subjects, as well as the fact that indirect observation may still have affected the work behavior of the observed subjects, the first thing which needs to be addressed, even in the conditions of a good utilization of available time for effective work (approximately 70%), is that relating to a particular low efficiency of such operations which was in the range of 8.6–9.0 h per double-pass operated hectare. From this point of view, the first hypothesis of this study was confirmed only from the productivity point of view. Indeed, there is limited information of operational performance metrics for this kind of jobs. Nevertheless, for something similar, de Oliveira et al. [11] found an efficiency rate of approximately 3.3 h per hectare which took 52% of the heart rate reserve during the effective work. The Romanian forestry-related rating system [31], on the other hand, indicates for the same job operational efficiencies in the range of 1.42–4.90 man-hours per 100 m^2^, which will probably ensure rest breaks-taking in a sustainable way. One way to improve the efficiency and to balance the effort given by workers would be that of deploying inter-row mechanized cultivation operations on two perpendicular directions since the plantation layouts would enable such an approach. In particular, this could contribute to a reduction of manually operated area to approximately one fourth compared to the current operational layouts.

In terms of physiological workload, worth mentioning that heart rate is a good estimator of the VO_2_ indicator [32] that is commonly used to predict the work difficulty in general ergonomic studies [21,22]. Cardiovascular workload, as found in this study indicates that this type of operation tends to overload the workers, therefore confirming the second hypothesis of the study. How the subjects experienced the workload was found to be related to their age. In average, the %HRR metric for the effective work was found to be very close to the threshold of 40% which, according to some authors [10], defines the limit between acceptable and unacceptable workloads. However, this outcome should be interpreted as indicative at least from two points of view. The first one refers to the impossibility to extend the findings to cohorts characterized by anthropometrics that are particularly contrasting to those which built the data from this study. The second one refers to the caution which should be used in the interpretation of data since the %HRR metric was based on the commonly accepted formula for estimating the maximum heart rate, which has its own limitations [33]. Also, an ambulatory trial found heart rates at rest lower when self-measured at home compared to those measured under expert observation [34]. Obviously, such an effect will probably lead to an underestimation of job’s difficulty, the same way the exposure to high thermal stress will. As such, and knowing the fact that the climate change affects the health of the workers [14], the thermal stress should be considered in the improvement of work in manual cultivation operations since the climate in the studied area is generally described by hot summers.

Most probably, an increased cardiovascular activity, as found in this study, is related to the type of work, work intensity and the body parts engaged in such work since the job tasks took a great deal of using handwork which is known to affect the heart rate response and characterizes the severity of muscular work [21]. Recovery time of heart rate is dependent on the exercise intensity and may reach more than 30 min [35,36], even if most of the recovery changes may occur in the first 1–2 min [36], while the heart rate response may be sensitive to postural changes [37]. For instance, switching from lying to sitting positions was found to increase the heart rate in some subjects by approximately 10 beats per minute [38]. Therefore, it was not surprising to find that for most of the subjects observed in this study the heart rate reserve was particularly increased also during the rest pauses and during the meal taking. It is difficult to place the manual cultivation operations, in terms of difficulty and risks, amongst other forestry jobs, given the fact that heart response is dependent on many factors such as the age of subjects, gender and their operational environment. Nevertheless, in motor-manual felling, estimates from the same flat-land area and for a worker having an age close to the average of this study [17] were close to those found in this study. In steep terrain forests of Turkey, for instance, harvesting and forest nursery work was found to be difficult to moderate jobs with heart rate reserves of approximately 41 and 32%, respectively [39], while jobs such as cable work in steep terrain [40,41] and cable rigging [42] may take more effort.

Work intensity was found to be light and moderate in most of the surveyed time (more than 95%), therefore partially confirming the third hypothesis of this study. Since ENMO values of up to 0.25 g are characterizing sedentary behaviors and light work such as standing still, dusting, sweeping the floor and self-paced walking [24], this intensity threshold was used to separate light intensity work in this study. In general, vigorous activity is considered to account for more than 21 mL × kg ^−1^ × min ^−1^ VO_2_ which roughly corresponds to accelerations corrected by the mean amplitude deviation of 0.45–0.5 g [25]. However, in this study, the intensity of work was considered to be moderate when ENMO had values from 0.25 to 1.0 g, by taking into account also the cardiovascular activity and the behavior or acceleration data in effective working events versus rest pauses. It should be mentioned that even in events such as the meal pauses, the subjects were not found to sit still all the time. 

Also, given the position in which the accelerometers were placed, the collected and analyzed data stands, in particular, for the activity of subjects’ back. This data may be correlated also with that coming from postural analysis where the back was found to be straight in 26% of the cases and bent, twisted or both in the rest of the cases. This outcome was partially consistent with the last hypothesis of this study. In this regard, the manual cultivation operation seems to be a job that does not require immediate postural redesign since the postural risk indexes were found to be less than 200% in most of the cases. However, the main problem here is that related to the back postures assumed by subjects which were particularly uncomfortable. Working predominantly with the back bent and twisted or bent forward and sideways (56.5% of the cases) may lead to health problems related to the lower back which is a known issue of forest operations jobs [14]. From this point of view, manual cultivation is a job that is even more hazardous compared to manual harvesting operations from Nordic countries [43] and close to that of motor-manual tree felling and processing operations from the area [17]. Compared to other kind of forestry-related partly mechanized jobs such as firewood processing [30] and wood debarking [44], manual cultivation seems to be riskier with the main problems coming from the back postures assumed during the work, since the arms and legs postures were found to be comfortable in most of the cases.

## 5. Conclusions

The main conclusion of this study is that the manual cultivation operations in poplar forests are rather difficult and hazardous, requiring reengineering tasks from ergonomic and public health points of view. To overcome the effect of small efficiency rates found in this study, mechanization should be extended by approaching the operated plots on two perpendicular directions, limiting this way the manual job to approximately one quarter compared to actual practices. Even if not documented by the available literature, such approaches are seldom used in the Romanian practice. Obviously, this way of extending the mechanized part of operations, will reduce also the continuous physical effort of the upper limbs and back by inter-placing movements from one tree to other, therefore it will lead to an increased use of bigger muscular groups and legs, that could help in attenuating the cardiovascular activity. By such measures, the intensity of manual work will be also decreased and the frequency of poor back postures will improve. Nevertheless, in such cases in which the approach of extending the mechanized operations is not feasible, a correct training of the workers, including a redesign of rest breaks could improve the status quo. These issues may be approached by intervention programs designed to tailor the work tasks, on the one hand, and to properly train the workers, on the other hand. Such approaches are important since the results of this study clearly indicated that the job in manual cultivation operations is characterized by a dynamic work that may overload the heart, upper limbs and the back of the workers. Also, incentives to attract youth in such operations should be developed and implemented at regional and national scale. 

## Figures and Tables

**Table 1:** Locations taken into study, study dates and weather condition during the study.

Forest District	Geographical Location	Forest Compartment and Area (Ha)	Observation Day	Weather Condition During the Study	Weed Height (M)	Abbreviation Used in This Study
Calafat	43°58′31.27″ N 22°54′04.42″ E	88D 0.76	13rd of June	T^1^ = 25.9 °C RH^2^ = 69.75%	0.7	L1 × 13
Segarcea	43°47′59.81″ N 23°36′01.88″ E	6C 2.00	18th of June	T^1^ = 22.0 °C RH^2^ = 76.75%	1.3	L2 × 18
Poiana Mare	43°50′50.12″ N 23°14′17.45″ E	70A 2.92	19th of June	T^1^ = 23.4 °C RH^2^ = 71.85%	0.5	L3 × 19
Poiana Mare	43°50′50.12″ N 23°14′17.45″ E	70A 2.92	20th of June	T^1^ = 23.6 °C RH^2^ = 69.33%	0.5	L3 × 20
Poiana Mare	43°50′50.12″ N 23°14′17.45″ E	70A 2.92	21st of June	T^1^ = 23.8 °C RH^2^ = 75.83%	0.5	L3 × 21
Calafat	43°58′31.27″ N 22°54′04.42″ E	88D 0.76	22nd of June	T^1^ = 19.8 °C RH^2^ = 85.00%	0.7	L1 × 22

^1^ T—air temperature. ^2^ RH—air relative humidity.

**Table 2:** Characteristics of the study group.

Subject	Abbreviation in This Study	Age (years)	Body Weight (kg)	Body Height (cm)	Body Mass Index
Subject 1	S1	36	100	186	28.91
Subject 2	S2	31	105	175	34.29
Subject 3	S3	40	110	180	33.95
Subject 4	S4	50	100	180	30.86
Subject 5	S5	47	71	176	22.92 ^1^
Subject 6	S6	40	70	165	25.71
Subject 7	S7	18	70	169	24.51 ^1^
Subject 8	S8	49	70	175	22.86 ^1^
Subject 9	S9	57	85	170	29.41
Subject 10	S10	50	68	165	24.98 ^1^
Subject 11	S11	67	67	170	23.18 ^1^
Subject 12	S12	62	75	179	24.41 ^1^
Subject 13	S13	45	70	173	23.39 ^1^
Subject 14	S14	57	102	180	30.79

^1^ Denotes normal weight according to Body Mass Index.

**Table 3:** Number of analyzed video files and frames.

Location and Observation Day	Number of Collected Video Files	Number of Frames Extracted for Postural Analysis of Each Worker	Number of Analyzed Frames	Number of Valid Frames
L2 × 18	13	1300	3900	1433
L3 × 19	18	1800	5400	2918
L3 × 20	16	1600	4800	3643
L3 × 21	17	1700	5100	2616
L1 × 13	8	800	2400	1657
L1 × 22	7	700	2100	946
Overall	79	7900	23,700	13,213

**Table 4:** Statistics of time consumption and estimates of work performance.

Subject, Location and Observation Day	Observation Time (h)	Effective Work Time (%)	Rest Time (%)	Meal Pause Time (%)	Delays (%)	Net Work Efficiency Rate (h/Ha)	Gross Work Efficiency Rate (h/Ha)
S1 × L2 × 18	4.8	61.80	26.91	9.86	1.43	26.295	29.599
S2 × L2 × 18	4.7	71.97	17.11	9.03	1.89	22.925	25.692
S3 × L2 × 18	4.5	74.84	13.04	11.66	0.46	23.159	26.352
S4 × L3 × 19	6.3	73.94	14.43	11.63	-	25.922	29.322
S5 × L3 × 19	6.2	57.36	28.44	11.61	2.59	25.074	29.234
S6 × L3 × 19	6.3	64.59	15.60	19.81	-	25.859	29.733
S7 × L3 × 20	5.7	75.18	14.49	10.20	0.13	21.695	24.163
S8 × L3 × 20	5.6	73.48	14.30	12.22	-	22.220	24.875
S9 × L3 × 20	5.5	74.44	14.60	10.96	-	22.044	24.754
S9 × L3 × 21	5.8	70.47	19.83	9.56	0.14	15.250	16.920
S4 × L3 × 21	5.8	52.77	37.18	9.92	0.13	14.981	17.497
S7 × L3 × 21	5.7	83.60	16.40	-	-	16.013	17.739
S10 × L1 × 13	3.4	73.85	24.65	-	1.50	69.148	69.711
S11 × L1 × 13	3.3	71.86	27.72	-	0.42	59.298	59.298
S12 × L1 × 13	3.4	77.54	22.46	-	-	59.438	59.438
S13 × L1 × 22	2.6	51.64	46.44	-	1.92	61.104	62.289
S12 × L1 × 22	2.5	83.89	15.58	-	0.53	50.118	50.699
S14 × L1 × 22	2.5	45.20	54.80	-	-	57.046	57.046
Overall	84.6	68.33	22.34	8.69	0.64	34.310	36.353

**Table 5:** Statistics of cardiovascular activity.

Subject, Location and Observation Day	Average Heart Rate (Bpm)	Heart Rate at Rest (Bpm)	Heart Rate Reserve for Effective Work	Heart Rate Reserve for Rest Pauses	Heart Rate Reserve for Meal Pauses	Overall Heart Rate Reserve
S1 × L2 × 18	108	50	44.00	42.98	37.19	42.95
S2 × L2 × 18	106	81	23.94	23.28	11.70	22.76
S3 × L2 × 18	104	69	32.06	33.24	23.37	31.25
S4 × L3 × 19	108	82	31.31	27.25	17.59	29.13
S5 × L3 × 19	117	87	38.95	33.77	13.89	34.56
S6 × L3 × 19	95	70	25.85	25.27	12.28	23.07
S7 × L3 × 20	105	67	30.64	25.23	16.53	28.40
S8 × L3 × 20	107	66	41.87	34.19	28.92	39.19
S9 × L3 × 20	102	71	32.99	35.70	35.10	33.62
S9 × L3 × 21	97	63	34.88	32.40	26.14	33.57
S4 × L3 × 21	100	78	25.08	23.94	12.86	23.45
S7 × L3 × 21	99	72	21.98	16.49	-	21.08
S10 × L1 × 13	114	80	39.26	31.42	-	37.24
S11 × L1 × 13	109	74	46.32	38.44	-	44.12
S12 × L1 × 13	112	67	51.23	45.67	-	49.98
S13 × L1 × 22	111	61	45.44	41.77	-	43.56
S12 × L1 × 22	109	70	44.17	42.38	-	43.85
S14 × L1 × 22	126	86	52.68	52.05	-	52.33
Overall	-	-	36.81	33.64	21.42	35.23

**Table 6:** Statistics of work intensity.

Subject, Location and Observation Day	Work Intensity Survey Time (h)	Share of Light Intensity Work (%)	Share of Moderate Intensity Work (%)	Share of High Intensity Work (%)
S1 × L2 × 18	4.7	38.96	55.93	5.11
S2 × L2 × 18	4.7	30.45	61.80	7.74
S3 × L2 × 18	4.5	33.25	59.71	7.05
S4 × L3 × 19	6.2	31.19	64.38	4.43
S5 × L3 × 19	6.2	54.01	43.76	2.22
S6 × L3 × 19	6.3	37.89	58.76	3.34
S7 × L3 × 20	5.7	33.00	62.43	4.57
S8 × L3 × 20	5.6	36.29	57.03	6.68
S9 × L3 × 20	5.5	23.64	74.05	2.31
S9 × L3 × 21	5.8	23.73	74.69	1.58
S4 × L3 × 21	5.8	44.44	53.43	2.12
S7 × L3 × 21	5.7	31.28	60.08	8.64
S10 × L1 × 13 ^1^	2.7 ^1^	15.80 ^1^	81.61 ^1^	2.59 ^1^
S11 × L1 × 13 ^1^	2.3 ^1^	96.24 ^1^	0.74 ^1^	3.02 ^1^
S12 × L1 × 13	3.3	18.61	71.67	9.72
S13 × L1 × 22	2.6	40.93	54.87	4.20
S12 × L1 × 22	2.5	20.57	75.06	4.37
S14 × L1 × 22	2.5	50.88	48.37	0.75
Overall ^2^	77.8 ^2^	34.59 ^2^	60.81 ^2^	4.60 ^2^

^1^ Denotes data that has not been used in the characterization of work intensity. ^2^ Averages computed by exclusion of data from ^1^.

**Table 7:** Share of back, arms and legs postures per codes described by OWAS.

Code	Share of Back Postures (%)	Share of Arms Postures (%)	Share of Legs Postures (%)
1	26.04	99.68	4.59
2	7.77	0.31	56.60
3	9.65	0.01	29.82
4	56.54	NA ^1^	3.71
5	NA ^1^	NA ^1^	3.48
6	NA ^1^	NA ^1^	0.17
7	NA ^1^	NA ^1^	1.63

^1^ Not applicable according to OWAS method.
